# Relative Bioavailability and Bioaccessibility and Speciation of Arsenic in Contaminated Soils

**DOI:** 10.1289/ehp.1003352

**Published:** 2011-07-13

**Authors:** Karen D. Bradham, Kirk G. Scheckel, Clay M. Nelson, Paul E. Seales, Grace E. Lee, Michael F. Hughes, Bradley W. Miller, Aaron Yeow, Thomas Gilmore, Sophia M. Serda, Sharon Harper, David J. Thomas

**Affiliations:** 1 National Exposure Research Laboratory, Office of Research and Development, U.S. Environmental Protection Agency, Research Triangle Park, North Carolina, USA; 2 National Risk Management Research Laboratory, Office of Research and Development, U.S. Environmental Protection Agency, Cincinnati, Ohio, USA; 3 Integrated Systems Toxicology Division, National Health and Environmental Effects Research Laboratory, Office of Research and Development, U.S. Environmental Protection Agency, Research Triangle Park, North Carolina, USA; 4 Science Advisory Board Staff Office, U.S. Environmental Protection Agency, Washington, DC, USA; 5 U.S. Environmental Protection Agency, Region 9, San Francisco, CA, USA

**Keywords:** arsenic, bioaccessibility, bioavailability, gastrointestinal, human health, human health risk assessment, metalloid, soil physicochemical properties, speciation

## Abstract

Background: Assessment of soil arsenic (As) bioavailability may profoundly affect the extent of remediation required at contaminated sites by improving human exposure estimates. Because small adjustments in soil As bioavailability estimates can significantly alter risk assessments and remediation goals, convenient, rapid, reliable, and inexpensive tools are needed to determine soil As bioavailability.

Objectives: We evaluated inexpensive methods for assessing As bioavailability in soil as a means to improve human exposure estimates and potentially reduce remediation costs.

Methods: Nine soils from residential sites affected by mining or smelting activity and two National Institute of Standards and Technology standard reference materials were evaluated for As bioavailability, bioaccessibility, and speciation. Arsenic bioavailability was determined using an *in vivo* mouse model, and As bioaccessibility was determined using the Solubility/Bioavailability Research Consortium *in vitro* assay. Arsenic speciation in soil and selected soil physicochemical properties were also evaluated to determine whether these parameters could be used as predictors of As bioavailability and bioaccessibility.

Results: In the mouse assay, we compared bioavailabilities of As in soils with that for sodium arsenate. Relative bioavailabilities (RBAs) of soil As ranged from 11% to 53% (mean, 33%). *In vitro* soil As bioaccessibility values were strongly correlated with soil As RBAs (*R*^2^ = 0.92). Among physicochemical properties, combined concentrations of iron and aluminum accounted for 80% and 62% of the variability in estimates of RBA and bioaccessibility, respectively.

Conclusion: The multifaceted approach described here yielded congruent estimates of As bioavailability and evidence of interrelations among physicochemical properties and bioavailability estimates.

The metalloid arsenic (As), a group 1 human carcinogen (International Agency for Research on Cancer 2004), is the second most common inorganic contaminant at Superfund sites [U.S. Environmental Protection Agency (EPA) 2001]. Hence, cancer risk associated with ingestion of As-contaminated soils (Calabrese et al. 1996; Davis et al. 1991; Dudka and Miller 1999) often drives risk assessments for human exposure to metal contaminants at Superfund sites (U.S. EPA 2007c). With increasing urbanization, exposure to As-contaminated soils grows more likely as residential areas extend into the vicinity or, in some cases, intrude onto Superfund sites (Scheckel et al. 2009). Reliable analysis of human health risks from ingestion of As-contaminated soil depends on estimating the bioavailability of As in the soil (U.S. EPA 1989). Current exposure estimates from ingestion of As-contaminated soils often do not consider differences between the bioavailability of As in water and soil (Ehlers and Luthy 2003). The use of default values that assume equivalent bioavailabilities for As in the two matrices can overestimate risk associated with ingestion of As-contaminated soil (Bradham and Wentsel 2010; U.S. EPA 2007b, 2007c). Speciation of As in soil, concentrations of other metals or metalloids, and other soil properties (e.g., pH and mineralogy) can affect the bioavailability of soil As and the amount available for systemic disposition [Kelly et al. 2002; National Research Council (NRC) 2003; U.S. EPA 2007b]. Because even small adjustments in soil As bioavailability estimates can significantly affect estimated risk and cleanup goals (U.S. EPA 2007c), methods are needed that quickly and inexpensively provide accurate and reliable data that can be applied to cleanups of As-contaminated sites worldwide.

Studies of soil As bioavailability have used species as diverse as rodents, swine, and monkeys (Casteel et al. 1997; Freeman et al. 1995; Lorenzana et al. 1996; Nagar et al. 2009; Ng et al. 1998; Pascoe et al. 1994; Rees et al. 2009; Roberts et al. 2002). Time and cost considerations may limit use of some species in bioavailability assays (U.S. EPA 2007a, 2007b). In the present study, we chose the mouse as the test species because of low purchase and husbandry costs, ease of handling, improved predictive value of data because of the feasibility of an increased sample size in assays, and the potential for widespread use of a mouse-based assay in many laboratories. Mice are well characterized physiologically and can be manipulated experimentally (e.g., altered dietary components, altered genotype) to determine the effects of biological variation on the gastrointestinal absorption of metals and metalloids. Extant data on gastrointestinal absorption of ingested arsenicals facilitate use of the mouse as a test species in assays of soil As bioavailability (Hughes et al. 2003, 2005, 2008). Although mice and humans differ in metabolism and disposition of arsenicals (Vahter 1999), similarities are sufficient to permit use of mouse data to create physiologically based pharmacokinetic models that can be scaled for humans (El-Masri and Kenyon 2008; Evans et al. 2008; Gentry et al. 2004a, 2004b; Hughes et al. 1999).

Use of complementary experimental approaches to assess bioavailability has been advocated as a strategy to develop models that reduce uncertainty in risk assessment (NRC 2003). In this study, we linked *in vivo* and *in vitro* assays with physicochemical characterization of soils in a unified approach to develop accurate and reliable methods for risk assessment of As-contaminated soils. Results for test soils and standard reference materials (SRMs) suggest that concerted use of *in vivo* and *in vitro* methods combined with physicochemical characterization of soils provides a stronger scientific basis for the refinement of risk assessments for As-contaminated soils. In addition, correlations between physicochemical properties of soils and estimates of As bioavailability and bioaccessibility indicate that use of physicochemical properties could profitably inform the refinement of both animal-based and *in vitro* assays.

## Materials and Methods

*Soil origin, processing, and physicochemical characterization.* For full description of soil origin, processing, and physicochemical characterization, see Supplemental Material (http://dx.doi.org/10.1289/ehp.1003352). Soils used in this study were collected from sites affected by mining and smelter activities. Physicochemical properties were determined in duplicate samples of each soil.

Arsenic speciation in soils was examined using the Materials Research Collaborative Access Team’s beamline 10-ID (Sector 10, Advanced Photon Source, Argonne National Laboratory, Argonne, IL). A principal component analysis coupled with linear combination fitting was used to identify the major As species in the samples. Linear combination fits were performed using X-ray absorption spectroscopy k^2^ space spectra from reference standards to As phases in the soil samples.

Arsenic concentrations in all soil and biological samples were determined by Instrumental Neutron Activation Analysis (INAA) at the Department of Nuclear Engineering, North Carolina State University (Raleigh, NC; mean As mass detection limit, 0.035 μg). All bioavailability and bioaccessibility calculations were based on INAA values.

*Mouse bioavailability assay.* The Institutional Animal Care and Use Committee of the U.S. EPA National Health and Environmental Effects Research Laboratory approved the protocol for mouse use, which assured humane treatment and alleviation of suffering. Female C57BL/6 mice 4–6 weeks of age (Charles River Laboratory, Raleigh, NC) were acclimated in groups of three in a 12/12-hr light/dark photocycle at 20–22°C. Mice had free access to rodent diet (TestDiet, Richmond, IN) and tap water that contained < 11 μg/L As (Kenyon et al. 2008). Composition of AIN-93G purified rodent diet (Reeves et al. 1993) obtained from Dyets (Bethlehem, PA) is given in Supplemental Material, Table 1 (http://dx.doi.org/10.1289/ehp.1003352). Soil-amended diets were prepared by thorough mixing of test soil with powdered AIN-93G purified rodent diet to a 1% (wt/wt) soil:diet ratio. Arsenate (As^V^)-amended diet prepared by addition of sodium arsenate heptahydrate (Sigma, St. Louis, MO) to powdered AIN-93G purified rodent diet was used to determine the bioavailability of a freely soluble As salt. Diets were stored at 4°C until used.

**Table 1:** Description, elemental composition, and As speciation in test soils.^*a*^

Arsenic speciation*b*						
Soil source*c*	Soil properties									As^V^			As^III^			Reduced chi squared*g*
	Soil ID	As*d* (mg/kg)	Fe*e,f* (g/kg)	Mn*e,f* (g/kg)	Al*e,f* (g/kg)	pH*f*	Sorbed As^V^ (%)	Scorodite (%)	Realgar (%)	Arsenopyrite (%)						
1		Urban residential		990		20.9		0.5		11.8		6.1		52.0		21.2		26.8		—		0.004
2		Urban residential		829		20.5		0.7		9.4		6.3		96.7		3.3		—		—		0.004
3		Urban residential		379		18.9		0.2		9.0		5.0		53.1		15.2		31.7		—		0.003
4		Smelter slag		837		294.4		2.7		13.2		7.2		18.7		1.6		47.7		32.1		0.001
5		Residential		244		46.0		0.8		21.7		7.3		96.2		3.8		—		—		0.002
6		Residential		173		63.4		0.7		20.9		6.6		66.8		33.2		—		—		0.002
7		Smelter slag		6,899		144.5		0.9		15.0		5.2		18.3		47.1		—		34.6		0.001
8		Residential		280		72.3		0.0		3.9		2.1		79.5		20.5		—		—		0.007
9		Smelter slag		4,495		120.1		0.4		12.3		2.6		67.6		32.4		—		—		0.011
10		NIST 2710		601		29.2		8.5		17.2		5.0		95.0		5.0		—		—		0.007
11		NIST 2710a		1,513		34.0		1.7		10.0		4.0		66.8		23.2		9.9		—		0.01
**a**The < 250 μm particle size fraction was used for all analyses. **b**Determined by linear combination of As X-ray absorption spectroscopy. **c**Source of As-contaminated soil. **d**Determined by INAA. **e**Extracted using U.S. EPA Method 3051A (U.S. EPA 2007d) and analyzed using U.S. EPA Method 6010C (U.S. EPA 2007e) by ICP-OES. **f**Data represent the mean of duplicate analyses. **g**Reduced chi-square values = (data – fit)^2^/data^2^.																						

At the start of an assay, three mice housed together during acclimation were transferred as a group to a metabolic cage that separated urine and feces (Nalgene, Rochester, NY). Twelve mice in four metabolic cages constituted an experimental run. Metabolic cages were maintained for 10 days under environmental conditions given above with unlimited access to test diet and drinking water. For sample collection and data analysis, the unit of observation was the cage and the standard assay for a soil had a sample size of four (except soil 9, which had a sample size of three). To examine assay variability and reproducibility, bioavailability of As in soils 4 and 10 were assayed two and three times, respectively, over a 2-year period.

Daily food consumption for each cage was calculated as the difference between the weight of the food hopper immediately after each morning’s filling and before replenishment the next morning. Cumulative food consumption for each cage was the sum of daily food consumption. Urine and feces were collected each morning from each metabolic cage. Combined body weights of the three mice in each metabolic cage were determined immediately before initial transfer into the metabolic cage and at termination. Mice were euthanized by carbon dioxide (CO_2_) anesthesia on day 10.

Daily urine or feces collections for each cage were stored at –20°C until processed to produce a single cumulative urine sample and single cumulative feces sample. After thorough mixing, multiple aliquots of the cumulative urine sample for each cage were taken for determination of As concentration by INAA. Cumulative urinary excretion of As was calculated as the product of As concentration in the cumulative urine sample and the volume of the cumulative urine sample. Cumulative feces samples were homogenized with a freezer/mill (model 6850; Spex CertiPrep, Metuchen, NJ). Multiple aliquots of cumulative feces sample were taken for determination of As concentration by INAA. Cumulative fecal excretion of As was calculated as the product of As concentration in the cumulative feces sample and the mass of the cumulative feces sample.

Absolute bioavailability (ABA) of As from ingestion of a soil- or As^V^-amended diet was calculated as the ratio of cumulative excretion of As in urine and cumulative dietary intake of As (NRC 2003; U.S. EPA 2007c). ABA is commonly calculated and expressed on a percentage basis:

%ABA = (cumulative As excreted in urine ÷ cumulative As consumed) × 100, [1]

with As measured in micrograms. Relative bioavailability (RBA) was calculated as the ratio of the ABA for As in a specific soil-amended diet to the ABA for As in a diet containing sodium arsenate (NRC 2003; U.S. EPA 2007c). RBA is commonly expressed on a percentage basis:

%RBA = (ABA of As in a specific diet ÷ ABA of As in sodium arsenate) × 100. [2]

*Bioaccessibility assays.* For a full description of bioaccessibility assays, see Supplemental Material (http://dx.doi.org/10.1289/ehp.1003352). Bioaccessible As was determined using an *in vitro* method developed by the Solubility/Bioavailability Research Consortium (SBRC) assay (Kelly et al. 2002). *In vitro* assays were performed in triplicate for each soil and included addition of 1 g test soil to 100 mL gastric fluid consisting of 0.4 M glycine at pH 1.5 in a 125-mL high-density polyethylene bottle and rotating end over end in a water bath at 37°C for 1 hr. All soils tested in the bioaccessibility protocol were identical to those administered to mice in the *in vivo* studies and used in the mineralogy studies described above. All *in vitro* extraction solutions were refrigerated at 4°C for preservation and subsequent analysis by Inductively Coupled Plasma–Optical Emission Spectroscopy (ICP-OES) (U.S. EPA 2007e).

*In vitro* bioaccessibility (IVBA) was calculated and expressed on a percentage basis:

%IVBA = (*in vitro* extractable mg As/kg soil ÷ total contaminant mg As/kg soil) × 100. [3]

*Statistical analysis.* Simple linear regression was used to evaluate the relationship between *in vivo* As RBA data and IVBA data and to examine the effect of selected soil physicochemical properties on As RBA and bioaccessibility. All analyses were performed using R software (version 2.9.1; R Development Core Team, Vienna, Austria), and figures were created using GraphPad Prism (version 5.0; GraphPad, San Diego, CA).

## Results

*Soil characterization.* Table 1 summarizes selected characteristics of test soils. Total As concentration in test soils ranged from 173 to 6,899 /mg/kg. Arsenic speciation by oxidation state varied among soils [see Supplemental Material, Figure 1 (http://dx.doi.org/10.1289/ehp.1003352)]. Soils 1, 3, 4, 7, and 11 had varying ratios of arsenite (As^III^) to As^V^ species; soils 2, 5, 6, 8, 9, and 10 contained only As^V^. We identified realgar in soils 1, 3, 4, and 11 and arsenopyrite in soils 4 and 7. Sorbed As^V^ and scorodite are common As species in soil environments and often result from the oxidation of As ore materials such as realgar or arsenopyrite. Concentration ranges of iron (Fe), manganese (Mn), and aluminum (Al) in soils were 18.9–294.4 g/kg, 0–8.5 g/kg, and 3.9–21.7 g/kg, respectively. Soil pH ranged from 2.1 to 7.3.

**Figure 1:**
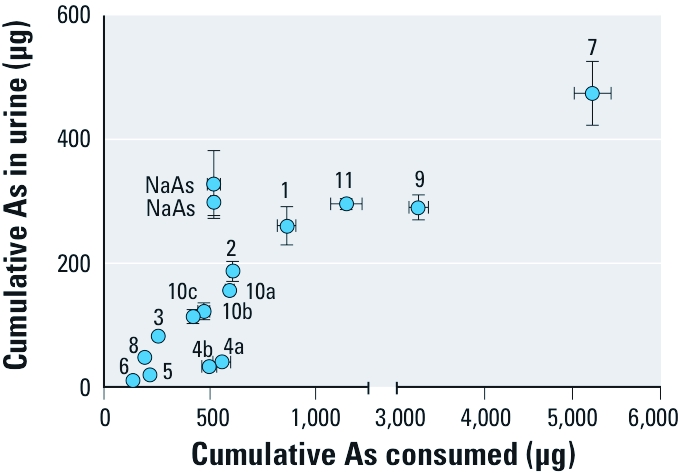
Relationship between cumulative As intake and cumulative urinary As excretion (mean ± SD). For soil numbers, see Table 1. Replicate assays are shown for soil 4 (4a, 4b) and soil 10 (10a, 10b, 10c). NaAs, sodium arsenate–amended diet.

*Mouse bioavailability assay.* The gross clinical condition of mice was unaffected by ingestion of any of the amended diets; amendment of diet with soil or sodium arsenate did not significantly affect cumulative diet consumption (data not shown). Thus, amendment of AIN-93G rodent diet with 1% (wt/wt) soil or As^V^ did not affect diet palatability for mice. Mean cumulative consumption of As strongly correlated with the concentration of As in the diet [see Supplemental Material, Figure 2 (http://dx.doi.org/10.1289/ehp.1003352)]. We evaluated mouse assay performance by determining the percentage of cumulative As intake recovered in cumulative urine and feces collections. Arsenic recoveries in excreta averaged 83.7% (range, 67–96%) for sodium arsenate–amended or soil-amended diets. For all dietary additives, percentage recovery and dietary As concentration were not correlated (*R*^2^ = 0.227; *p* = 0.398, Pearson product moment correlation).

**Figure 2:**
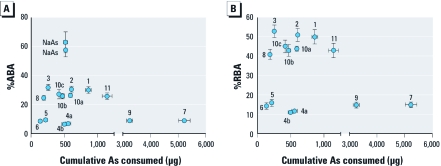
%ABA (*A*) and %RBA (*B*) of As from amended diets as a function of cumulative As intake (mean ± SD). Replicate assays are shown for soil 4 (4a, 4b) and soil 10 (10a, 10b, 10c); NaAs, sodium arsenate–amended diet.

Increasing cumulative ingestion of As from amended diets was associated with increasing cumulative urinary excretion of As (Figure 1). Figure 2A shows As ABA estimates from diets amended with As^V^, test soils, or SRMs. Duplicate assays with As^V^-amended diet yielded an As ABA of approximately 60%. Arsenic ABA estimates for test soils ranged widely from approximately 7% to approximately 33%. Duplicate assays with diets amended with soil 4 (4a, 4b) yielded As ABA estimates of 6.7% and 7.1%. Triplicate assays with diets amended with National Institute of Standards and Technology’s NIST-2710, Montana Soil SRM (soils 10a, 10b, 10c), yielded As ABA estimates ranging from 25.9% to 27.2%. For comparison, NIST-2710a SRM-amended diets (soil 11) dosed at multiple levels yielded an As ABA of approximately 26% for each dose level [see Supplemental Material, Figure 2 (http://dx.doi.org/10.1289/ehp.1003352)]. Figure 2B shows As RBA estimates for test soils and SRMs. Relative to As^V^ bioavailability, As RBA estimates for test soils ranged from 11% to 53%. Arsenic RBA estimates for NIST 2710–amended diet (soil 10) and NIST 2710a–amended diet (soil 11) were approximately 44%. Supplemental Material, Table 2, summarizes data from mouse assays.

**Table 2:** Results of linear regression analyses to explore the influence of select soil properties on As RBA and IVBA.

RBA					IVBA				
Predictor	Equation	*R*^2^	*p*-Value	Equation	*R*^2^	*p*-Value
Sorbed As^V^ (%)		0.2*x* + 17.1		0.14		0.26		0.3*x* + 18.4		0.11		0.31
Scorodite (%)		–0.4*x* + 38.9		0.10		0.35		–0.7*x* + 50.9		0.16		0.22
Realgar (%)		0.1*x* + 31.1		0.01		0.80		0.2*x* + 36.1		0.01		0.73
Arsenopyrite (%)		–0.7*x* + 36.2		0.28		0.09*		–0.7*x* + 42.5		0.16		0.23
AsV (%)		0.2*x* + 19.0		0.05		0.50		0.1*x* + 26.9		0.02		0.70
As^III^ (%)		–0.2*x* + 34.7		0.05		0.50		–0.1*x* + 40.2		0.02		0.70
As (mg/kg)		*x* + 37.3		0.17		0.21		*x* + 45.2		0.15		0.23
Fe (g/kg)		–0.1*x* + 43.5		0.48		0.02**		–0.2*x* + 51.4		0.32		0.07*
Al (g/kg)		–1.9*x* + 57.3		0.34		0.06*		–2.7*x* + 73.3		0.32		0.07*
Mn (g/kg)		0.7*x* + 31.0		0.01		0.77		1.1*x* + 36.3		0.01		0.76
pH		–2.2*x* + 43.3		0.05		0.52		–1.2*x* + 44.0		0.01		0.82
Fe+Al (mol/kg)		–8.8*x* + 48.7		0.58		0.01^#^		–10.5*x* + 57.9		0.40		0.04**
Log(Fe+Al) (mol/kg)		–53.1*x* + 41.6		0.80		0.00^#^		–67.5*x* + 50.1		0.62		0.00^#^
*p ≤ 0.10, **p ≤ 0.05, and ^#^*p* ≤ 0.01.												

**Figure 3:**
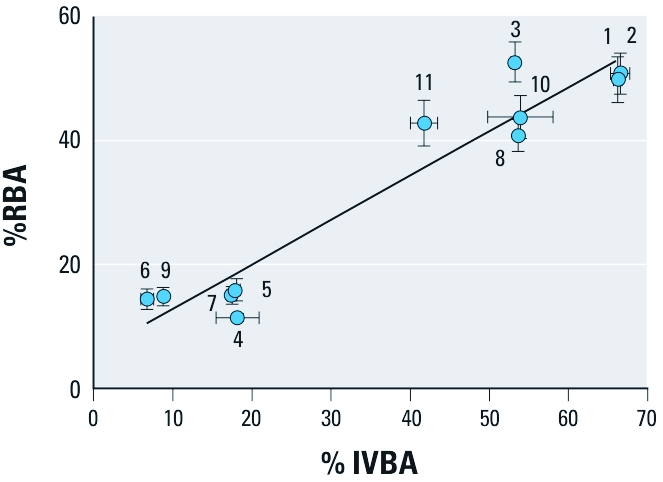
Correlation between estimates of As bioaccessibility and bioavailability (mean ± SD). %RBA = 0.72(%IVBA) + 5.64 (*R*^2^ = 0.92).

*Correlations among estimates of bioaccessibility and bioavailability and physicochemical properties.* IVBA values ranged from 6.8% to 67% (SD were 0–3%). We extracted NIST SRMs (soils 10 and 11) multiple times over the course of the study in accordance with the SBRC assay (SDs were 4.1 and 1.7, respectively). We used linear regression to assess predictability of As RBAs from bioaccessibility values derived from the SBRC assay. The derived regression model accounted for 92% of the variability in As bioavailability observed in the mouse assay (*R*^2^ = 0.92; Pearson correlation = 0.96; Figure 3).

We examined predictability of As bioavailability or bioaccessibility from the physicochemical properties and speciation of As in soils by simple linear regression (Table 2). Physicochemical properties of soil that were significant predictors (*p* < 0.10) of As RBA estimates were also significant predictors of IVBA estimates, with the exception of percent arsenopyrite. Among predictors, sums of concentrations of extractable soil Fe and Al (Fe+Al) accounted for the largest amount of variation in RBA and IVBA estimates (*R*^2^ = 0.58 and 0.40, respectively). Log(Fe+Al) improved the predictive value of this term (*R*^2^ = 0.80 and 0.62 for RBA and IVBA, respectively). Although multivariable linear regression analysis has been used to estimate As bioavailability (Yang et al. 2002), application of this method in the present study did not materially improve predictions of As RBA or IVBA.

## Discussion

The concordance of RBA and bioaccessibility estimates obtained in mouse and *in vitro* assays with common physicochemical characteristics of soils suggested that these approaches could be used in a complementary manner to reduce uncertainty in assessment of risk associated with exposure to As-contaminated soils.

The mouse assay proved adaptable for use with soils with a wide range of As concentrations and physicochemical properties. Amended diets were palatable, and as anticipated from earlier studies (Xie et al. 2004), mice remained in apparent good health throughout the experimental period. In this study, calculation of the As ABA used results from the mouse assay for a diet amended with 7 ppm As as sodium arsenate. This amendment produced As dose levels of 8.9 and 9.2 mg/kg in duplicate studies [see Supplemental Material, Tables 1 and 2 (http://dx.doi.org/10.1289/ehp.1003352)]. The dose levels for As^V^-amended diets exceeded those for contaminated soils 3, 5, 6, 8, and 10b; approximately equaled (i.e., with overlapping standard deviations) those for soils 4a, 4b, 10a, and 10c; and was lower than those for soils 1, 2, 7, 9, and 11. Hence, for most soils tested, the concentration of As^V^ added to the diet equaled or exceeded that present in diet after soil amendment. Although additional studies with As^V^-amended diets are needed to confirm that estimates of bioavailability of As^V^ or As in soil are unaffected by As concentration in amended diets, studies in As^V^-treated laboratory mice suggest that dose level does not affect the rate of urinary clearance of As (Hughes and Thompson 1996; Hughes et al. 1994; Kenyon et al. 2008). Similarities in the pattern and extent of urinary clearance of As in mice that have received sodium arsenate over a wide range of dose levels suggest that dose level does not influence uptake of As^V^ across the gastrointestinal barrier or its clearance into urine. In the absence of a change in the rate of urinary clearance of As over a wide dose range, it is likely that mice ingesting diets amended with As^V^ or As-containing soils will reach whole-body steady-state body burden during the experimental period used in this study (Hughes et al. 2003).

Similar estimates of As bioavailability obtained for soils 4 and 10 in assays over a 2-year period indicated that assay performance was stable (Figure 2A,B). In adult female mice receiving repeated daily oral doses of sodium arsenate, the body burden of As reaches steady state after 8 or 9 days of dosing (Hughes et al. 2003, 2010). Under steady-state conditions, concentrations of As in tissues and outputs of As in urine and feces will reach plateau values that will remain unchanged throughout the dosing interval. Although concentrations of As in urine and feces are both good indicators of current exposure, the predominance of urine as the route for As clearance after oral administration of inorganic As (Hughes et al. 2003) makes it ideal for estimating the extent of absorption of dietary As. Summing amounts of As excreted in urine and feces during the experimental period can be used to approximate recovery of As in the mouse assay. For the materials evaluated in the mouse assay, recoveries of ingested As in excreta ranged from 67% to 96%. However, these values should be regarded as minimal estimates because they do not include As that is retained in tissues of mice.

The mouse assay can be further refined by examining the role of dietary composition on the estimates of soil As bioavailability obtained with this model. Compared with AIN-93 purified diets, the human diet common in developed countries derives more calories from fat, contains less fiber, and may not be optimal in terms of mineral and vitamin composition. These differences in dietary composition could affect the bioavailability of As in two ways. First, the elemental composition of the diet can affect As uptake across the gastrointestinal barrier. For example, an increasing concentration of phosphate reduces *in vitro* uptake of As^V^ by Caco-2 intestinal cells derived from human colonic adenocarcinoma cells (Calatayud et al. 2010) and gastrointestinal uptake of As in rats dosed orally with As^V^ (Gonzalez et al. 1995). Second, in humanized gnotobiotic mice the microbiota of the gastrointestinal tract is quickly altered by consumption of a diet with a high fat and high sugar content (Turnbaugh et al. 2009). Alteration of the microbiota of the gastrointestinal tract produced by changes in dietary composition could alter gastrointestinal uptake of ingested As^V^. Recent studies show that the anaerobic microbiota from the mouse cecum extensively metabolize As^V^ to produce inorganic thioarsenicals and methylated oxy- and thioarsenicals (Pinyayev et al. 2011). The mouse model can readily be adapted to examine effects of dietary composition of diets on the bioavailability of As in soils.

Soil As RBA estimates obtained in juvenile swine and monkeys have ranged from 0% to 52% (Casteel et al. 1997; Freeman et al. 1995; Lorenzana et al. 1996; Rees et al. 2009; Roberts et al. 2002; Rodriguez et al. 1999). Comparisons of As RBA data obtained in mice and juvenile swine are problematic because of differences in experimental design and dosing levels. However, four soils have been evaluated in both species. For three soils (soils 9, 10, and 11 in this study), As RBA estimates from mouse and juvenile swine differed by 4%, 0%, and 1%, respectively (U.S. EPA 2009). For the fourth soil (soil 8 in this study), As RBA estimates differed by 19.1% (with estimates of 40.9% for mouse and 60% for juvenile swine. Differences in As RBAs for mouse and juvenile swine may reflect physiological differences between species. Additional soils should be evaluated in both species to identify possible sources of variability and permit a detailed comparison of the assays.

A recent NRC report has recommended development and validation of *in vitro* assays that can replace *in vivo* assays and can provide reliable and accurate data that reduce uncertainty in risk assessment (NRC 2007). This recommendation prompted development of bioaccessibility assays that reflect processes that control As bioavailability in the human gastrointestinal tract (Basta et al. 2007; Juhasz et al. 2007; Kelly et al. 2002; Rodriguez et al. 1999; Ruby et al. 1999). High correlation (*R*^2^ = 0.92, Pearson correlation = 0.96) between the As bioaccessibility data from the SBRC assay and As RBA estimates from the mouse assay is consistent with the high correlation of estimates of As RBA from juvenile swine with As bioaccessibility estimates from the SBRC assay (*R*^2^ = 0.75, Pearson correlation = 0.87) (Juhasz et al. 2009). The correlation of findings from the SBRC assay and the mouse assay suggests that the bioaccessibility assay provides useful information about the characteristics of As-containing soils that influence As RBA as measured in the mouse assay. In addition, strong agreement of estimates from the SBRC *in vitro* assay and the mouse assay suggest that the mouse assay can be used to validate performance of bioaccessibility assays.

Metal speciation and the concentrations of Fe, Al, and Mn are known to affect solubilities and bioavailabilities of metals in soils (Bradham et al. 2006; Kelly et al. 2002; NRC 2003; Scheckel et al. 2009). In this study, we evaluated the effects of As speciation and metal concentrations on estimates of soil As RBA and bioaccessibility obtained in the mouse assay and SBRC assay by linear regression analyses. We found significant inverse correlations between concentrations of extractable Fe and Al in soils and estimates of soil As RBA and bioaccessibility. For example, the log-transformed sum of Fe+Al accounted for 80% and 62% of the variability in estimates of As RBA and bioaccessibility, respectively. The high predictive value of log(Fe+Al) suggests that sorption of As to Fe and Al oxides reduces As solubilization and thereby reduces As RBA and bioaccessibility. Beak et al. (2006a, 2006b) found similar results for As bioaccessibility using a modified Rodriguez et al. (1999) *in vitro* method, which investigated As sorption on ferrihydrite [Fe^3+^_5_O_3_(OH)_9_] and corundum (Al_2_O_3_). Thus, determination of the concentrations and forms of Fe and Al in soils may be useful in assessing As bioavailability. Several clay minerals contain ferrous and ferric iron that, upon release via weathering, will form iron oxides and hydroxides in soil environments (Bowell 1994). Similar processes are also identified for aluminum and manganese oxides in soils (Jenne 1968; McKeague et al. 1971). Lower As RBA estimates for soils containing sulfide forms of As (realgar or arsenopyrite) may reflect slow dissolution kinetics of these mineral species. Although arsenopyrite was present in only two of the test soils, its presence significantly reduced As bioavailability estimates (*p* < 0.10). This finding is consistent with reports showing that As in arsenopyrite is bound tightly; therefore, As bioavailability is likely to be low (Roberts et al. 2007). Additional studies would be useful to identify other metals and metalloids in soils that are potential modifiers of As bioavailability and bioaccessibility and to determine concentration dependencies of these interactions.

## Conclusions

A multifaceted approach combining *in vivo* assays, *in vitro* assays, and physicochemical characterization of soils yielded comparable estimates of As bioavailability and provided evidence of interrelations among physicochemical properties and estimates of As bioavailability. The range of As RBA estimates in this study (11–53%) implies that use of a default value of 100% for As bioavailability in human health risk assessments may overestimate risk associated with exposure to As-contaminated soils. Further studies with the mouse assay and the *in vitro* assay coordinated with physiochemical characterization of test soils can confirm and extend the results obtained in this study and identify refinements in experimental design and data analysis that can improve the accuracy and reliability of estimates of bioaccessibility and bioavailability.

## Supplemental Material

(192 KB) PDF
