# Supplementary material for: Relative Bioavailability and Bioaccessibility and Speciation of Arsenic in Contaminated Soils
Source: Environ Health Perspect. 2011 Jul 13;119(11):1629–34. doi: 10.1289/ehp.1003352 (PMC3226497; doi:10.1289/ehp.1003352)
Supplement: (192 KB) PDF [file ehp.1003352.s001.pdf]

Supplemental Material:
Relative Bioavailability and Bioaccessibility and Speciation of Arsenic in Contaminated Soils

Karen D. Bradham [*,1], Kirk G. Scheckel[2], Clay M. Nelson[1], Paul E. Seales[3], Grace E. Lee[3], Michael F. Hughes[3], Bradley W. Miller[2], Aaron Yeow[4], Thomas Gilmore[1], Sharon Harper[1], David J. Thomas[3]

[1] U.S. Environmental Protection Agency, Office of Research and Development, National Exposure Research Laboratory, Research Triangle Park, North Carolina 27711

[2] U.S. Environmental Protection Agency, Office of Research and Development, National Risk Management Research Laboratory, Cincinnati, Ohio 45224

[3] U.S. Environmental Protection Agency, Office of Research and Development, National Health and Environmental Effects Research Laboratory, Integrated Systems Toxicology Division, Research Triangle Park, North Carolina 27711

[4] U.S. Environmental Protection Agency, Science Advisory Board Staff Office, 1200 Pennsylvania Avenue NW, Washington, District of Columbia 20460

*Corresponding author: bradham.karen@epa.gov, 109 TW Alexander Drive, MD-D205-05, Research Triangle Park, North Carolina; phone: (919) 541-9414; fax: (919) 541-3527; e-mail: bradham.karen@epa.gov.

Table of Contents

Methods..3
Figure 1..6
Table 1..7
Table 2..8
Figure 2..9
Figure 3..10
References..11

METHODS

Soil processing and physicochemical characterization - Soils used in this study were dried (< 40°C) and sieved to < 250 μm. The U.S. EPA considers particles < 250 μm most likely to adhere to hands and be subsequently ingested by hand-to-mouth contact, especially in young children (U.S. EPA 2007a, 2007b). Soil samples were homogenized, riffled, and aliquots were split for each of the participating labs by procedures described in Blume et al. (1991).

Selected extractable inorganics in each soil were determined by extracting the < 250 μm soil using U.S. EPA Method 3051A (2007c) with analysis by Varian Vista-MPX Inductively Coupled Plasma-Optical Emission Spectrometer (ICP-OES) according to U.S. EPA Method 6010C (2007d). Soil pH was determined using 1:1 soil:water suspension using a combination pH electrode (Thomas 1996).

Arsenic speciation in soils was examined using the Materials Research Collaborative Access Team's (MRCAT) beamline 10-ID, Sector 10 at the Advanced Photon Source (APS), Argonne National Laboratory (ANL), Argonne, IL. The electron storage ring operated at 7 GeV in top-up mode. A liquid N_2 cooled double crystal Si(111) monochromator was used to select incident photon energies and a platinum-coated glass mirror was used for harmonic rejection. The beam energy was calibrated by assigning the first derivative inflection point of the L_{III}-absorption edge of gold metal (11919 eV) foil. Three As K_{α} (11867 eV) X-ray absorption spectroscopy (XAS) spectra were collected in fluorescence mode (16-element solid state Ge detector, Canberra) at room temperature for every soil and reference sample. Data analysis was conducted using IFEFFIT software (Ravel and Newville 2005). Replicate scans for each sample were merged, then normalized, and converted into *k* space. A principal component analysis coupled with linear combination fitting (LCF) was used to identify the major As species in the

samples. Linear combination fits (LCF) were performed using XAS k^2 space spectra from reference standards to As phases in the soil samples. Reference materials for LCF, based on principal component analysis, included arsenate sorbed to ferrihydrite (Sorbed As^V), scorodite [$Fe(As^VO_4)$], realgar ($As^{III}S$), and arsenopyrite ($FeAs^{III}S$). Data for LCF fits reveal As speciation in each soil as ratios of these mineral forms.

Mouse Bioavailability Assay - Female C57BL/6 mice (four to six weeks old) were purchased from Charles River Laboratory (Raleigh, NC). During acclimation, these mice were housed in groups of three in polycarbonate cages with cellulose bedding and environmental enhancements (e.g., cardboard tubes) in a 12 hour light–12 hour dark photocycle at 20-22 ^oC. Mice had free access to rodent diet (TestDiet, Richmond, IN) and sipper-type water bottles containing tap water filtered on-site with inline sand and charcoal filters and re-chlorinated to a final concentration of 3 to 5 ppm Cl.

The basal diet was AIN-93G purified powdered rodent diet (Dyets, Bethlehem, PA), which is formulated to support growth, pregnancy, and lactation in mice (Supplemental Material, Table 1) (Reeves et al. 1993). Soil-amended diets were prepared by thorough mixing of 10 g of test soil with 990 g of AIN-93G purified rodent diet with a goal of a 1% (w/w) soil:diet ratio. A sodium arsenate-amended diet prepared by addition of sodium arsenate heptahydrate (Sigma, St. Louis, MO) to AIN-93G purified rodent diet was performed in duplicate to determine the bioavailability of a freely soluble As salt for comparison with bioavailabilities of As in different soils. All diets were stored at 4 ^{o}C until used.

Mice were placed in metabolic cages on the morning of day 1 with free access to diet and drinking water for 9 days. On the mornings of day 2 through 10, urine and feces were collected

from each metabolic cage. Food hoppers were removed on the afternoon of day 9 and mice were fasted overnight with free access to drinking water until the morning of day 10.

For each cage, daily urine or feces collections were pooled to create cumulative urine and feces samples. These samples were stored in freezers at -20 ^{o}C until processed. Pooling of urine and feces collections over the experimental period yielded a single sample for each metabolic cage. The pooled urine sample for each cage was thawed at room temperature and its volume determined. This sample was well mixed and multiple aliquots were taken for determination of the concentration of As by INAA (mean As mass detection limit of 0.035 µg).

Bioaccessibility assays - Bioaccessible As was determined using an in vitro method developed by the Solubility/Bioavailability Research Consortium (SBRC method) for comparison with in vivo data (Kelly et al. 2002). All soils tested in the bioaccessibility protocol were identical to those administered to mice in the in vivo and mineralogy studies described above. In vitro procedures were conducted by adding one gram of test soil to 100 mL of buffered glycine solution (0.4 M glycine, pH 1.5) in a 125 mL HDPE bottle, rotating end-over-end in a water bath at 37 ^{o}C (body temperature) for one hour. Extractions were performed in triplicate for each test soil. Blanks, National Institute of Standards and Technology (NIST) Standard Reference Material (SRM) soils (Montana soils 2710 and 2710a), and spikes were analyzed to meet quality assurance and quality control requirements. All labware were cleaned, washed, and rinsed with deionized water prior to use according to a standard protocol. Analysis of the extracts by ICP-OES included QA/QC procedures as described above. The method detection limit (MDL) in extraction fluid was calculated to be 0.1 mg L^{-1} for Method 6010C.

Normalized XANES spectra **Derivative of the XANES spectra**

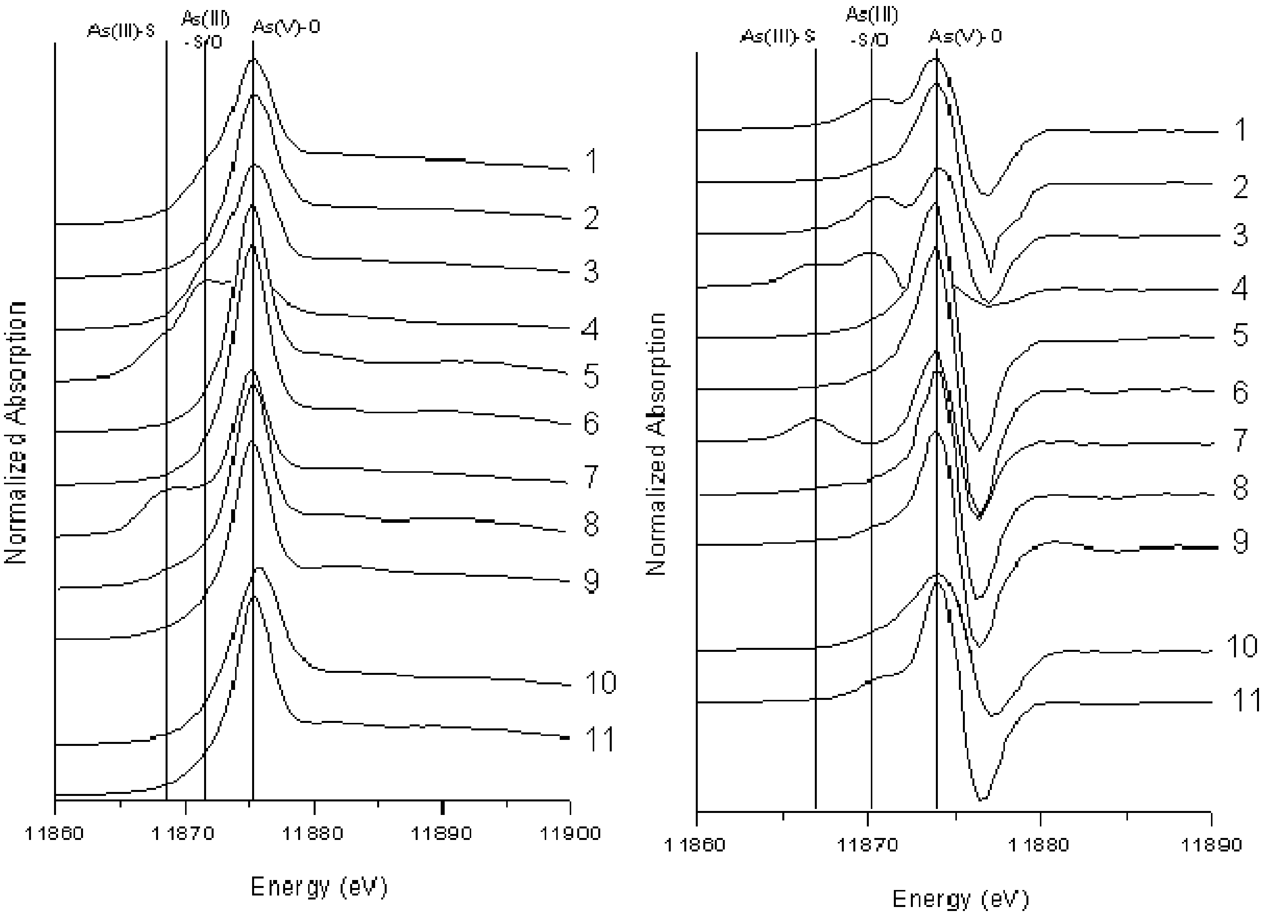

Supplemental Material, Figure 1. Normalized and derivative of the XANES spectra. Soils 1 through 3 were collected from urban residential sites. Soils 4, 7, and 9 are slag soils from smelter sites. Soils 5, 6, and 8 came from non-urban residential sites. Soil 10 is NIST 2710 and soil 11 is NIST 2170a.

Supplemental Material, Table 1. Composition of basal AIN-93G purified rodent diet[a]

Ingredient	Grams per kilogram	Kilocalories per kilogram
Casein	200	716
Sucrose	100	400
Cornstarch	397.486	1430.9496
Dyetrose	132	501.6
L-cystine	3	12
Cellulose	50	0
Soybean oil	70	630
t-Butylhydroquinone	0.014	0
Mineral mix	35	30.8
Vitamin mix	10	38.7
Choline bitartrate	2.5	0
	1000	3760.0496

a. Composition of AIN-93 G purified rodent diet provided by Dyets (Bethlehem, PA) is in accordance with recommendations of the American Institute of Nutrition Ad Hoc Writing Committee of the reformulation of the AIN-76A rodent diet (Reeves et al., 1993).

Supplemental Material, Table 2. Summary data on arsenic consumption, dosage level, excretion, and bioavailability for materials evaluated in mouse assays[a].

Dietary Amendment	Cumulative As consumption (µg)	As dosage (mg/kg)	Cumulative As Excretion (% of dose)			% Bioavailability	
			Urine	Feces	Sum	Absolute	Relative[b]
Sodium arsenate #1	518.0<br>30.4	9.2<br>0.5	63.0<br>7.2	11.6<br>2.2	74.6<br>6.6	63.0<br>7.2	100
Sodium arsenate # 2	517.7<br>14.2	8.9<br>0.3	57.8<br>4.8	8.3<br>1.7	66.1<br>3.3	57.8<br>4.8	
Soil							
1	862.8<br>43.5	15.8<br>0.9	30.1<br>2.3	47.5<br>6.4	77.6<br>4.1	30.1<br>2.3	49.9<br>3.7
2	606.9<br>14.3	11.9<br>0.1	30.8<br>2.0	36.6<br>1.8	67.4<br>2.8	30.7<br>2.0	50.9<br>3.3
3	256.2<br>12.3	5.3<br>0.2	31.6<br>1.9	56.1<br>0.7	87.7<br>2.4	31.9<br>2.0	52.8<br>3.2
4A	556.0<br>41.1	10.4<br>0.8	7.1<br>0.3	81.6 16.1	88.8<br>15.8	7.1<br>0.3	11.8<br>0.6
4B	496.8<br>36.1	10.4<br>0.5	6.7<br>0.3	83.3<br>3.1	89.9<br>3.2	6.7<br>0.3	11.1<br>0.6
5	217.3<br>3.3	4.1<br>0.2	9.6<br>1.1	68.9<br>1.5	78.5<br>1.7	9.6<br>1.1	15.9<br>1.8
6	136.0<br>4.0	2.6<br>0.1	8.7<br>1.0	87.6<br>3.7	96.3<br>2.8	8.7<br>1.1	14.4<br>1.6
7	5224.1<br>204.5	101.7<br>2.2	9.1<br>0.8	78.8<br>2.4	87.9<br>3.0	9.1<br>0.8	15.0<br>1.4
8	191.8<br>9.2	3.9<br>0.1	24.8<br>1.6	56.8<br>1.1	81.6<br>1.3	24.7<br>1.6	40.9<br>2.6
9	3252.0<br>109.4	60.3<br>3.3	8.9<br>0.9	79.5<br>4.7	88.4<br>3.8	9.0<br>0.9	14.8<br>1.5
10A	593.3<br>20.6	10.1<br>0.2	26.4<br>1.3	54.1<br>1.9	80.5<br>0.6	26.5<br>1.3	43.8<br>2.2
10B	419.2<br>11.5	6.5<br>0.3	27.1<br>3.2	54.9<br>2.8	82.0<br>4.0	27.2<br>3.2	45.0<br>5.3
10C	470.9<br>27.8	9.5<br>0.7	25.9<br>1.8	55.6<br>1.4	81.5<br>0.5	25.9<br>1.8	42.9<br>3.0
11	1144.7<br>73.8	23.7<br>1.0	25.8<br>2.2	57.8<br>2.1	83.6<br>3.5	25.9<br>2.2	42.9<br>3.7

a – Mean (upper) and standard deviation (lower). Sample size of 4 for all soils except soil 9 where sample size is 3.

b - Relative % bioavailability for soils calculated as: (absolute % bioavailability for soil)/mean absolute % bioavailability for sodium arsenate). Here, mean absolute % bioavailability for sodium arsenate was 60.4.

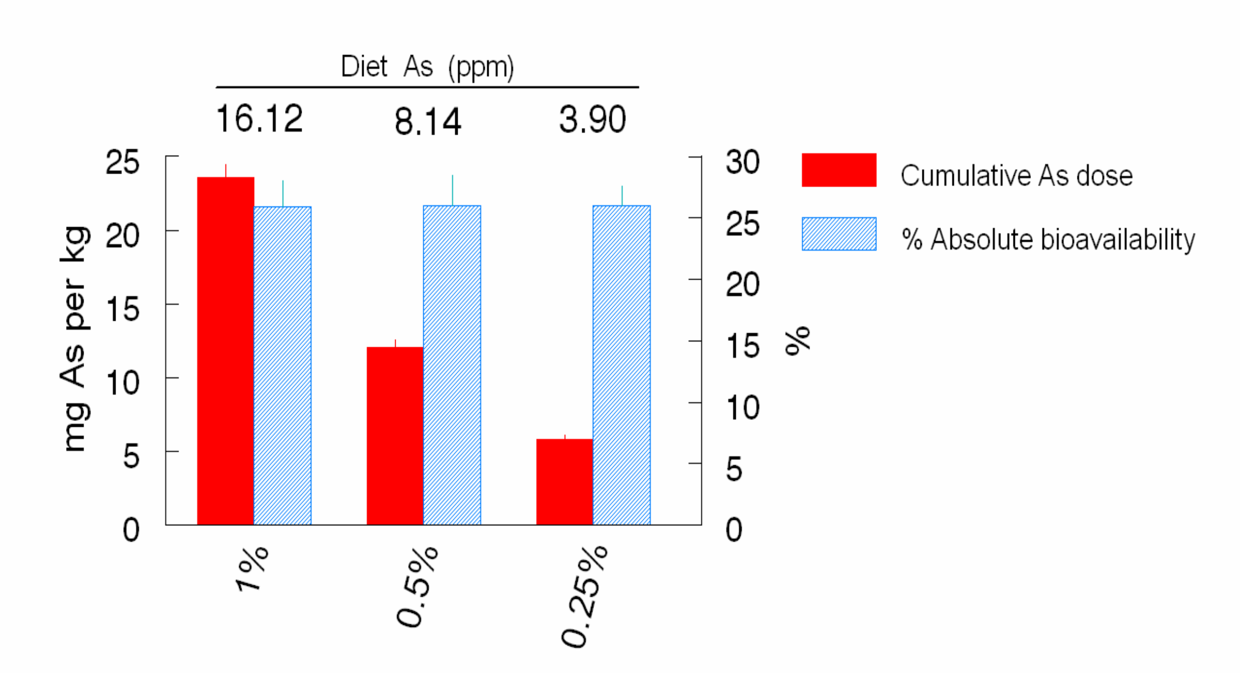

Supplemental Material, Figure 2. Diets amended by adding by weight 1, 0.5, or 0.25% NIST-2710a soil to the powdered purified rodent chow resulted in mice receiving arsenic at multiple dosage levels over approximately a 4-fold range. Estimates of absolute bioavailability of arsenic from this soil were the same for each dosage level and estimation of absolute bioavailability was independent of dosage level.

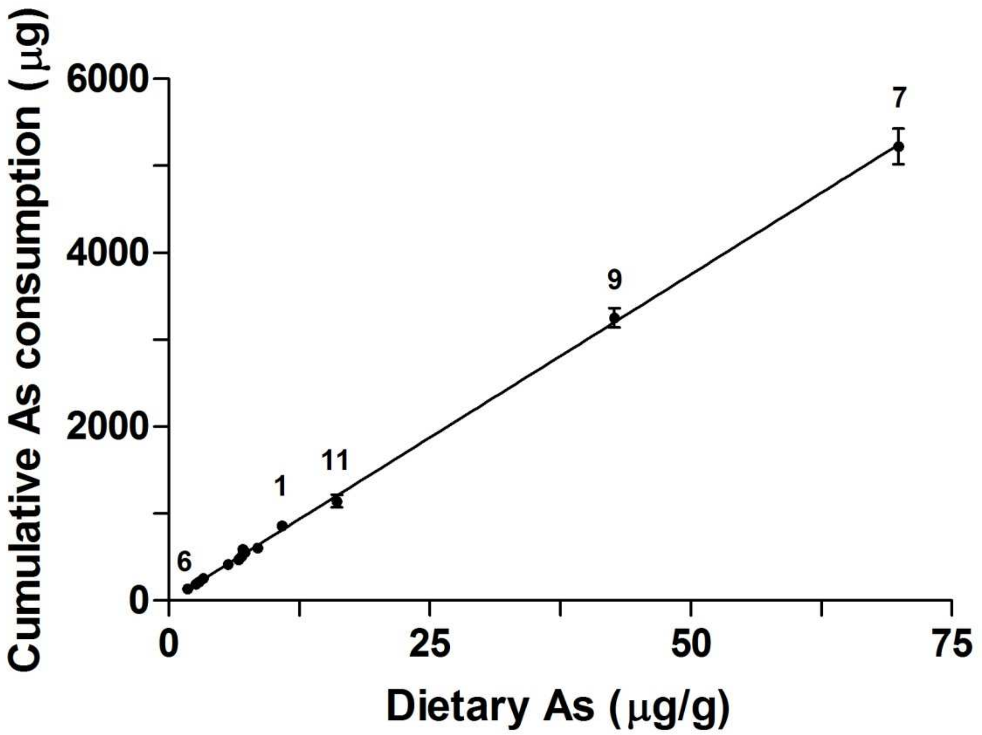

Supplemental Material, Figure 3. Relationship between concentrations of As in mouse diet and mean cumulative consumption of As over the experimental period. Equation for linear regression ($y = 75.38x - 4.7644$, $r^2 = 0.999$).

REFERENCES

Blume LJ, Schumacher BA, Schaffer PW, Cappo LA, Papp ML, Van Remortel RD, et al. 1991. Handbook of Methods for Acid Deposition Studies, Laboratory Analyses for Soil Chemistry. EPA/600/S4-90/023. Las Vegas, NV:U.S. EPA.

Kelly ME, Brauning SE, Schoof RA, Ruby MV. 2002. Assessing Oral Bioavailability of Metals in Soil. Columbus:Battelle Press.

Ravel B, Newville M. 2005. ATHENA, ARTEMIS, HEPHAESTUS: data analysis for X-ray absorption spectroscopy using IFEFFIT. J Synchrotron Rad 12:537-541.

Reeves PG, Nielsen FH, Fahey GC. 1993. AIN-93 purified diets for laboratory rodents: final report of the American Institute of Nutrition ad hoc writing committee on the reformulation of the AIN-76A rodent diet. J Nutr 123:1939-1951.

U.S. EPA (U.S. Environmental Protection Agency). 2007a. Framework for Metals Risk Assessment. EPA 120/R-07/001. Washington, DC:US EPA.

U.S. EPA (U.S. Environmental Protection Agency). 2007b. Estimation of Relative Bioavailability of Lead in Soil and Soil-like Materials Using in Vivo and in Vitro Methods. OSWER 9285.7-77. Washington, DC:U.S. EPA, Office of Solid Waste and Emergency Response.

U.S. EPA (U.S. Environmental Protection Agency). 2007c. Method 3051A: Microwave Assisted Acid Digestion of Sediments, Sludges, Soils, and Oils. In: Test Methods for Evaluating Solid Waste, Physical/Chemical Methods. SW-846. Washington, DC:U.S. EPA, Office of Solid Waste.

U.S. EPA (U.S. Environmental Protection Agency). 2007d. Method 6010C: Determination of Inorganic Analytes by Inductively Coupled Plasma-Atomic Emission Spectrometry. In: Test Methods for Evaluating Solid Waste, Physical/Chemical Methods. SW-846. Washington, DC:U.S. EPA, Office of Solid Waste.
